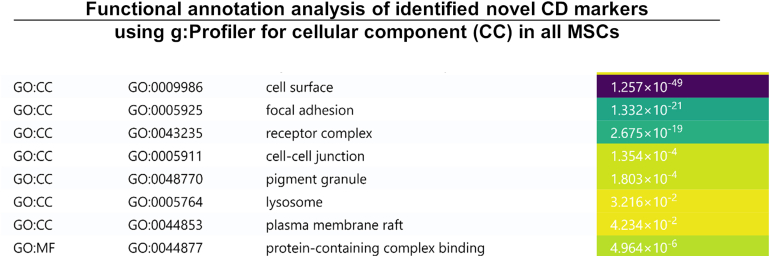# Chondrogenic potential of mesenchymal progenitors from somatic and cartilage-derived iPSCs is predicted by their transcriptomic signatures

**DOI:** 10.1016/j.gendis.2025.101730

**Published:** 2025-06-22

**Authors:** Nazir M. Khan, Thanh N. Doan, Jarred M. Kaiser, Hicham Drissi

**Affiliations:** a Department of Orthopaedics, Emory University, Atlanta, GA 30329, USA; b Joseph Maxwell Cleland, Atlanta VA Medical Center, Decatur, GA 30033, USA

**Keywords:** Cartilage, Chondrogenesis, Gene regulatory network, Hypertrophy, iPSC, MSC, Non-classical surface markers, TGFβ

## Abstract

Mesenchymal stem cells (MSCs) are widely used in regenerative therapy but face limitations like low abundance, replicative senescence, donor variability, and restricted plasticity. Induced pluripotent stem cell-derived MSCs (iMSCs) may provide an alternative, but their similarities or intrinsic differences with adult MSCs remain unknown. This study compares the chondrogenic potential of iMSCs derived from chondrocyte-specific induced pluripotent stem cells, with bone marrow-derived MSCs, adipose-derived stem cells, and dedifferentiated chondrocytes. Chondrogenic differentiation was performed in high-density pellet cultures with short-term or long-term TGFβ3 treatment. Chondrogenic gene arrays, gene regulatory networks, and gene ontology analysis revealed divergent signaling pathways. Bulk RNA sequencing was performed to characterize the transcriptomic profiles of each MSC. Results showed that iMSCs produced cartilage with hyaline-like features and minimal hypertrophy, distinguishing them phenotypically from adult MSCs. Gene regulatory network analyses identified EGF, FGFR, FLT1, and HIFA as iMSC hub genes for chondrogenic differentiation. Molecular signaling analysis unveiled that TGFβ3 induced SMAD2/3, not SMAD1/5, suppressing hypertrophy in iMSC chondrogenesis. RNA sequencing highlighted cell-specific differences, functional heterogeneity, and divergent cell signaling profiles between iMSCs and adult MSCs. Using integrated transcriptome and proteome analyses, we identified and validated eight novel non-classical CD markers that may help further characterize MSCs and potentially discriminate iMSCs from other cell types. This study further advanced our understanding of MSC behaviors, emphasizing the importance of origin-specific considerations and refining the molecular description of iMSCs as an unlimited source of chondroprogenitors for cartilage regeneration.

## Introduction

Mesenchymal stem cells (MSCs) are a promising cell type for the repair of mesenchyme-derived tissues, such as focal cartilage defects,^1^ with over 200 clinical studies reporting the use of MSCs for cartilage repair.^2^ MSCs can participate in cartilage repair either directly by supporting chondrogenic differentiation or indirectly by regulating the metabolism and immune function of the host. Yet, the regenerative potential of MSCs may be heavily dependent on their tissue of origin. While MSCs were originally isolated from bone marrow (BM), MSCs have also been isolated from several other somatic tissues, including adipose tissue,^1^ cord blood,^3^ trabecular bone,^4^ and synovium.^5^ BM-MSCs have widely been studied and used for cartilage regeneration despite their well-documented limitations, including restricted proliferative capability, replicative senescence after *in vitro* expansion, invasive and painful isolation procedures, and low isolation yield, which impedes their widespread clinical applications.^1^ Consequently, adipose tissue-derived stem cells (ADSCs) have emerged as a viable MSC source due to their ease of harvest, abundance, and extensive capacity for self-renewal, but have also demonstrated a lower efficiency of differentiation than BM-MSCs.^6^

Differentiation of MSCs towards a chondrogenic fate requires stimulation with various factors, regardless of the cell source. While growth factors like transforming growth factor-beta (TGFβ) or bone morphogenetic protein-2 (BMP2) effectively promote chondrogenesis in BM-MSCs and ADSCs, they also induce chondrocyte hypertrophy and promote terminal chondrocyte differentiation,^7^^,^^8^ hindering the overall success of MSC-based cartilage repair and regeneration.^7^^,^^9^ The successful generation and integration of *de novo* hyaline native cartilage remains a clinical challenge in part due to an inability to curtail the hypertrophy of adult somatic MSCs.^10^^,^^11^ Induced pluripotent stem cells (iPSCs) may be an alternative cell source for generating articular cartilage.^12^, ^13^, ^14^, ^15^, ^16^, ^17^, ^18^, ^19^ Recent studies have demonstrated low levels of hypertrophy in chondrocytes derived from iPSCs.^20^^,^^21^ Unlike adult somatic MSCs, which exhibit declining proliferative and differentiation capabilities after multiple passages, iPSC-derived MSCs (iMSCs) display an unrestricted proliferative capacity. These key characteristics could be leveraged through current manufacturing practices to generate patient-specific iMSCs for successful cartilage regeneration. Yet, there has not been a comprehensive and systematic comparative study among iMSCs and the two most used somatic adult MSCs, BM-MSCs and AD-MSCs.^22^^,^^23^

MSCs derived from different tissue sources have demonstrated diverse, dissimilar, and heterogeneous behavior.^24^^,^^25^ MSCs suffer from poor definition, as the criteria for MSC identification proposed by the International Society for Cellular Therapy (ISCT) lack consistency across different tissue sources.^26^ Growing evidence indicates that these criteria do not uniformly apply across diverse tissues and species, as they only define general functional and morphological characteristics.^27^ A clear and unambiguous definition of MSCs and their cell-surface markers is critical to ensure a pure population of cells for a highly repeatable and highly reliable manufacturing process.

Thus, our current study had two goals. We first comprehensively compared the *in vitro* chondrogenic differentiation potential of iMSCs with BM-MSCs and ADSCs, providing mechanistic insights into their discrepant and heterogeneous responses. For this comparative study, we used iMSCs derived from chondrocyte-specific iPSCs (AC-iPSCs), which we previously generated from normal human articular chondrocytes.^14^^,^^28^ AC-iPSCs were chosen over skin fibroblast-derived iPSCs (SF-iPSCs) based on our prior findings that AC-iPSCs exhibited enhanced chondrogenic potential compared with SF-iPSCs.^28^ In the second goal, we aimed to enhance the accuracy of MSC identification across various origins through an unbiased transcriptomic and proteomic characterization of these MSCs. Together, our data clearly define a unique transcriptomic signature for each of the progenitor cells tested, providing new grounds for future correlations between the cell source and the chondrogenic and/or chondroprotective capacity of each MSC product.

## Materials and methods

### iPSCs culture and derivation of mesenchymal progenitors (iMSCs)

We used our previously reported iPSCs derived from healthy articular chondrocytes (AC-iPSCs)^14^^,^^28^ These iPSCs were generated using commercially acquired normal human knee articular chondrocytes (#CC-2250, NHAC-kn, Lonza, Walkersville, Maryland, USA) isolated from a 47-year-old male Caucasian donor. The iPSC colonies were cultured using mTeSR™1 media (StemCell Technologies) in a 6-well culture plate coated with 0.1% Geltrex® (Peprotech).^14^^,^^15^ For routine culture and maintenance, iPSC colonies were passaged after reaching 70% confluency by washing once with 1 × phosphate-buffered saline solution and treating with ReLeSR™ reagent (StemCell Technologies) into a new 6-well plate using mTeSR™ 1 medium supplemented with 10 μM Rho-associated kinase (ROCK) inhibitor Y-27632 (StemCell Technologies).

Derivation of mesenchymal progenitors (iMSCs) was performed using our established direct plating method.^13^, ^14^, ^15^^,^^29^ Briefly, iPSC colonies were treated with accutase and then plated onto gelatin-coated culture plates in the presence of iMSC growth medium consisting of Dulbecco's modified Eagle medium (DMEM)-high glucose (Gibco), 10% defined fetal bovine serum (Hyclone), 1% nonessential amino acids, 1 × penicillin-streptomycin, and 5 ng/mL recombinant human basic fibroblast growth factor (rhbFGF; Peprotech).^14^^,^^15^ After 2–3 passages, cells adopted a homogenous spindle-shaped morphology and adhered to the culture plate without any coating. We have previously demonstrated that these iMSCs possessed all the criteria of MSCs as defined by the ISCT.^14^ We used early-passage (<10) iMSC cultures for chondrogenic differentiation to avoid any potential effect of higher passage and increased doubling time on the differentiation process.

### Flow analysis for the surface markers in iMSCs

We performed phenotyping analysis for the MSC surface markers proposed by the ISCT.^30^ Surface markers of MSCs were labeled and analyzed using anti-human antibodies against cluster of differentiation 73 (CD73), CD95, CD105, CD44, CD45, CD31, and CD29 ^14,31^. Isotype-matched controls were employed to discern nonspecific fluorescence. Cell acquisition was performed using BD FACSAria™ and operated with FACS Diva software (Becton–Dickinson). In each analysis, a minimum of 20,000 cells was acquired, and the data were analyzed using FlowJo Software.^14^^,^^31^

### Adult somatic stem cell culture

The MSCs from bone marrow were obtained from Lonza (Catalogue number #PT-2501) (donor #1, 21-year-old male, lot #6F3502; donor #2, 31-year-old male, lot #19TL155677; and donor #3, 22-year-old female, lot #7F3674). Similar adipose-derived stem cells were purchased from Lonza (Catalogue number #PT-5006) (donor #1, 23-year-old female, lot #18TL212639; donor #2, 25-year-old male, lot #41348; and donor #3, 51-year-old female, lot #0000439846). All MSCs passed the company's quality inspection for cell viability, adipogenic and osteogenic differentiation, and cell surface markers. Somatic MSCs were cultured in mesenchymal stem cell basal medium (MSCBM) supplemented with MSCGM SingleQuots kit (Lonza medium) and maintained at 37 °C in a humidified atmosphere containing 5% CO_2_ per manufacturer's instructions. Medium was changed twice per week, and cells were split when they reached 80%–90% confluency. Somatic MSCs were used at an early passage (≤3 passages) for all chondrogenic pellet culture.

Purchased healthy human articular chondrocytes were used as a control (NHAC-kn, Lonza, Walkersville, Maryland, USA). Articular chondrocytes were expanded using the chondrocyte growth medium (CGM, containing 5% fetal bovine serum, 0.1% gentamicin sulphate-amphotericin B, 0.5% b-FGF, 0.2% R3-IGF-1, 0.2% insulin, and 0.1% transferrin) at 37 °C and 5% CO_2_. After reaching 80% confluency, the cells were split up to three passages and then used for the differentiation experiments. During passaging of cells in 2D monolayer culture, articular chondrocytes were dedifferentiated into a fibroblast-like phenotype and termed as dedifferentiated chondrocytes (DDCs).^32^ These DDCs were re-differentiated into chondrocytes using pellet culture.

All human cells were obtained from commercial sources (Lonza, USA) as de-identified biospecimens collected under their institutional review board-approved protocols with informed donor consent. This study did not involve identifiable data or human subject interaction and thus did not require institutional review board approval.

### Chondrogenic differentiation of iMSCs and adult somatic MSCs

Chondrogenic differentiation of iMSCs, BM-MSCs, ADSCs, and DDCs was performed in high-density culture conditions using a 3D pellet.^14^^,^^15^ Briefly, a single-cell suspension of MSCs was prepared using 0.25% trypsin-ethylenediaminetetraacetic acid (EDTA), and 0.25 × 10^6^ cells were transferred into 15-mL polypropylene tubes and centrifuged at 300 *g* for 5 min to pellet the cells, which were then cultured in 500 μL chondrogenic induction medium (hMSC chondrogenic differentiation BulletKit™ with SingleQuots, Lonza). The pellets were supplemented with continuous or transient TGFβ3 stimulation. In the continuous treatment group, pellets were cultured in chondrogenic medium supplemented with 10 ng/mL TGFβ3 (Peprotech, Rocky Hill, NJ) for 21 days. In the transient TGFβ3 treatment group, pellets were maintained in TGFβ3-supplemented chondrogenic medium for the first 7 days and then cultured in chondrogenic medium without TGFβ3 supplementation for an additional 14 days. Pellets without supplementation of TGFβ3 served as a control. During the differentiation process, the tube lid was left open to allow gas exchange. The pellets were incubated at 37 °C under a humidified atmosphere with 5% CO_2_ for 21 days, and the differentiation medium was changed every two days. Pellets were harvested at 7, 14, and 21 days of chondrogenic differentiation.

### RNA isolation, reverse transcription, and gene expression analysis

RNA was isolated from iPSCs, iMSCs, somatic MSCs, and DDCs at day 7, 14, and 21 using TRIzol reagent (Invitrogen).^33^^,^^34^ Reverse transcription was performed using a high-capacity cDNA synthesis kit (Thermo Fisher) following the manufacturer's instructions. Quantitative polymerase chain reaction (qPCR) was performed using PowerUp™ SYBR® Green master mix (Applied Biosystems). All PCR experiments were performed with three biological replicates from each group and two technical replicates. Melt curve analysis was performed for the specificity of the primer and the authenticity of the amplicon. The mRNA expression of chondrogenic and hypertrophy genes was normalized to β-actin mRNA, and relative expression levels were calculated using the 2^−ΔΔCT^ method.^33^^,^^34^

### Chondrogenic gene array analysis

We employed an array of 88 genes involved in chondrogenic differentiation, extracellular matrix (ECM) organization, cartilage development, and signaling pathways associated with BMP and TGFβ receptor signaling. The targeted primer sets (realtimeprimers.com, #HOST-I) allowed for a more thorough and focused examination of genes pertinent to chondrogenic differentiation and facilitated a more straightforward interpretation of data than a genome-wide characterization.

The human chondrogenic PCR array consisted of 88 primer sets for chondrogenic genes and 8 housekeeping gene primers, including actin-beta (*ACTB*), beta-2-microglobulin (*B2M*), glyceraldehyde-3-phosphate dehydrogenase (*GAPDH*), glucuronidase-beta (*GUSB*), hypoxanthine phosphoribosyltransferase 1 (*HPRT1*), phosphoglycerate kinase 1 (*PGK1*), peptidylprolyl isomerase A (*PPIA*), and ribosomal protein L13a (*RPL13A*). RNA (2 μg) was reverse-transcribed into cDNA, and the PCR array was collected in a 20 μL volume with 1 × SYBR® Green, 2 μL cDNA, and 1 μL primers at a concentration of 500 nM in 96-well plates. The reaction was run on a 7500 PCR system (Applied Biosystems™) with the following protocol: 95 °C for 5 min, followed by 50 cycles at 95 °C for 10 s and 58 °C for 45 s. Ct values for all genes were exported to the PCR Array Quant-HK norm spreadsheet analysis template, and expression levels were calculated using the ΔΔCt method, assuming primer efficiency remained consistent across the gene panel. Data normalization utilized the median value of the expression of the 8 housekeeping genes. The fold change in gene expression was determined by dividing the normalized expression in the experimental group by that in the control group. The differentially expressed genes (DEGs) were visualized by a heatmap generated using the ClustVis web tool.^35^ Significant differences between groups were assessed using a student's *t*-test.

### Gene ontology and gene regulatory network (GRN) analysis

DEGs during chondrogenic differentiation of iMSCs and somatic MSCs were analyzed using STRING (Search Tool for the Retrieval of Interacting Genes) for the functional enrichment of gene ontology terms, focusing on biological processes.^36^ The significance of enriched pathways and corresponding *p*-values were determined through the cumulative hypergeometric *t*-test, with false discovery rate applied.

Subsequently, we conducted a GRN analysis to identify the hub genes associated with the regulation of chondrogenic differentiation in iMSCs, BM-MSCs, ADSCs, and DDCs. Co-expression network analysis was performed on the most significant DEGs during the 21-day chondrogenic differentiation in the continuous TGFβ3 group for all four cell types. The resulting network was visualized using Cytoscape (version: 3.7.1) as outlined in our previous studies.^37^^,^^38^ GRNs containing genes strongly associated with each other were identified through module analysis using the Molecular Complex Detection Algorithm (MCODE) plugin (version: 1.5.1) within Cytoscape.^39^ Gene lists of the most significant modules in this GRN were extracted, and gene ontology term analysis was performed to retrieve their biological processes that modulate the chondrogenic differentiation in each cell type.

### Library preparation and bulk RNA sequencing of iMSCs and adult somatic cells

To characterize the transcriptome profiles in their native states, iMSCs, BM-MSCs, ADSCs, and DDCs were collected for bulk RNA sequencing. RNA from all samples was extracted using miRNeasy kits, with on-column DNase digestion to eliminate genomic DNA contamination. The quality of the extracted RNA was assessed using Nanodrop. RNA integrity was evaluated using the Agilent 2200 Bioanalyzer. Libraries were generated from 250 ng RNA utilizing the TruSeq Stranded Total RNA Sample Prep Kit (Illumina), employing the Poly A enrichment method. Subsequently, sequencing was executed using the NovaSeq PE 150 system at the Novogene UC Davis Sequencing Center.

### Preprocessing of bulk RNA-sequencing data

We assessed error rates and GC (guanine and cytosine nucleotides) content distribution of the raw data. Subsequently, data was filtered to eliminate reads of low quality or those containing adaptors. Clean reads were then aligned to the human reference genome (GRCh38). We used the DESeq2 method to analyze DEGs. Pairwise gene expression levels were computed using FPKM (fragments per kilobase of transcript sequence per million base pairs sequenced) values. The calculation of fold change in gene expression was carried out on the filtered datasets, utilizing normalized signal values.

### Differential gene expression, cluster dendrogram, principal component analysis, and interaction network analysis of RNA-sequencing data

DEGs were identified using DESeq2 in R Bioconductor.^40^ Significance was set to *p* < 0.01 and log_2_ fold-change > 2 with a multiple testing correction for false discovery rate. Principal component analysis of the DEGs was performed in R-Bioconductor.^14^^,^^38^^,^^41^^,^^42^ During data preprocessing, rows underwent unit variance scaling, and principal components were computed using singular value decomposition with imputation. For cluster dendrogram analysis, rows were centered, unit variance scaling was applied, and columns were clustered using the correlation distance and average linkage method. MSC-specific gene signatures were identified by analyzing the genes exclusively present in a specific cell type with an FPKM value > 5.0.

To assess transcriptomic similarity among the four cell types based on MSC markers, a similarity matrix heatmap was generated using FPKM values for known MSCs through the Clustergrammer web tool.^43^ Venn diagram analysis was performed to compare the common genes among iMSCs versus adult MSCs or DDCs using Venny2.0. Furthermore, genes common to all four cell types were subjected to functional annotation analysis to determine the enrichment of “Reactome pathways” and gene ontology terms for biological processes and cellular components using STRING (version: 11.0).

### Identification of novel MSC markers based on integrated analysis of transcriptome and proteome data

RNA-sequence data were analyzed for the expression of all known CD marker genes. Expression values were normalized using the FPKM method. For the comparison of CD markers, a Venn diagram network was generated to visualize the shared and unique CD genes among the four MSCs using EVenn, an online web tool.^44^

Liquid chromatography/mass spectrometry-based proteomics data in BM-MSCs available in the public domain were used for analyzing the protein expression of CD markers.^45^ For this analysis, raw data files were downloaded from the ProteomeXchange Consortium (http://proteomecentral.proteomexchange.org) using dataset identifier “PXD001856”, and differentially abundant proteins were analyzed by comparing the protein levels of BM-MSCs and ESCs. The ProteomicsDB (https://www.proteomicsdb.org) database was used for validating the identified CD markers by quantifying the protein expression of surface proteins in MSCs.^46^

### Statistical analysis

Data were presented as mean ± standard deviation of at least three biological replicates (independent donors) unless otherwise specified (*n* = 3 biological donors). Biological replicates for BM-MSCs and ADSCs were defined as independently sourced cells from three different human donors per group, each handled in separate experimental runs. Each biological replicate was assessed in three technical replicates per experimental condition. All experiments were repeated at least three times. Statistical comparisons among three or more groups were performed using one-way ANOVA, followed by Tukey's Honest Significant Difference post-hoc analysis, using GraphPad Prism (v10.1.1). Statistical significance was set at *p* < 0.05.

## Results

### Generation of iMSCs and characterization of mesenchymal nature

We used our previously established approach for the generation of iMSCs as outlined in Figure 1A. After multiple passages (*n* ≤ 3), the iMSCs acquired spindle-shaped fibroblast-like morphology and attained plastic adherence ability (Fig. 1A). We measured mRNA expression of markers of pluripotency, mesenchymal, and known MSC surface markers using reverse transcription qPCR assay. Our gene expression analysis showed a gradual decrease in expression of the pluripotency markers octamer-binding protein 4 (*OCT4*), nanog homeobox (*NANOG*), SRY-box transcription factor 2 (*SOX2*), and Krüppel-like factor 4 (*KLF4*) from iPSC to iMSC stages (Fig. 1B). Additionally, gene expression of mesenchymal markers, such as runt-related transcription factor 1 (*RUNX1*), twist family bHLH transcription factor 1 (*TWIST1*), *CD44*, and collagen type I alpha 1 (*COL1A1*), gradually increased their expression levels throughout mesodermal differentiation. iMSCs expressed high levels of other MSC surface marker genes, such as *CD29*, *CD44*, *CD73*, *CD90*, *CD105*, *CD166*, *CD108*, and *CD130* (Fig. 1B). Furthermore, flow cytometric analysis showed that these iMSCs displayed a robust expression of the surface markers CD105, CD166, CD90, CD44, and CD29 in line with the standard criteria of ISCT for MSCs (Fig. 1C). Thus, our direct plating method generated iMSCs that displayed all the markers of MSCs and exhibited mesenchymal gene expression.

**Figure 1:**
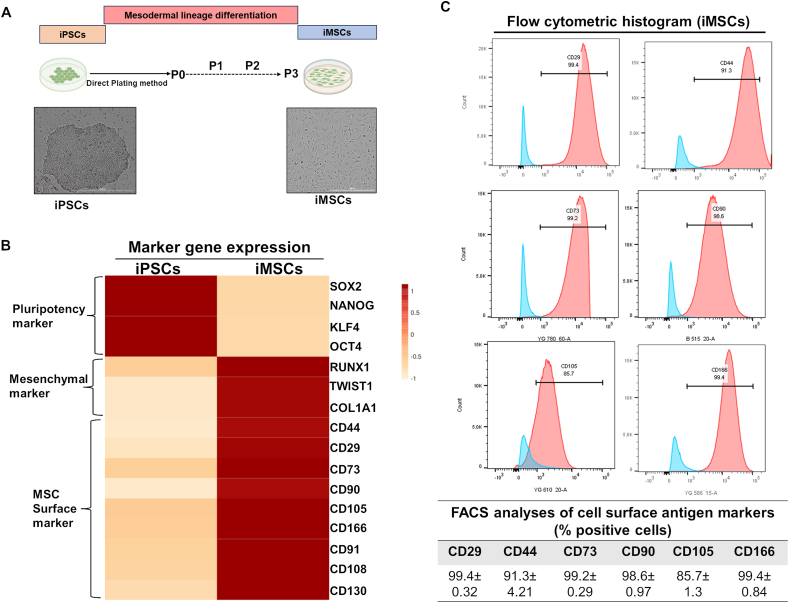
Generation of iMSCs and characterization of mesenchymal nature. **(A)** Schematic representation of the differentiation of iPSCs toward mesenchymal progenitors using the direct plating method. The morphology of iMSCs derived from iPSCs shows elongated, spindle-shaped cells. Representative images are shown for iMSCs at passage 3. Scale bar, 100 μm. **(B)** The heatmap shows the gene expression (fold change) as analyzed by quantitative PCR assay for pluripotency markers and mesenchymal markers in iPSCs and iMSCs. Quantitative PCR analysis showed significant suppression of pluripotency marker genes *OCT4*, *NANOG*, *KLF4*, and *SOX2* and induction of mesenchymal genes *TWIST1*, *COL1A1*, and *RUNX1* in iMSCs relative to parental iPSCs. β-actin served as the housekeeping gene and internal control. Expression data is represented as fold change relative to the respective parental iPSCs. **(C)** Expression of surface antigens in iMSCs by flow analysis. Representative flow cytometric histogram shows that iMSCs express markers associated with the mesenchymal phenotype (positive for CD29, CD44, CD73, CD90, CD105, and CD166) (*n* = 3). The red histogram shows the antibody-stained population, and the blue profile shows the negative isotype-stained population. Results were from one representative experiment (*n* = 3). MSC, mesenchymal stem cell; iPSC, induced pluripotent stem cell; iMSC, iPSC-derived MSC; OCT4, octamer-binding protein 4; NANOG, nanog homeobox; SOX2, SRY-box transcription factor 2; KLF4, Krüppel-like factor 4; RUNX1, runt-related transcription factor 1; TWIST1, twist family bHLH transcription factor 1; COL1A1, collagen type I alpha 1; CD, cluster of differentiation.

### Chondrogenic potential of iMSCs and adult somatic MSCs

We next evaluated the chondrogenic differentiation potential of iMSCs in comparison with adult somatic MSCs. As MSC chondrogenesis is influenced by factors such as timing, dose, and duration of TGFβ3 exposure,^47^^,^^48^ we examined two treatment conditions: continuous and transient TGFβ3 stimulation (Fig. 2A).

**Figure 2:**
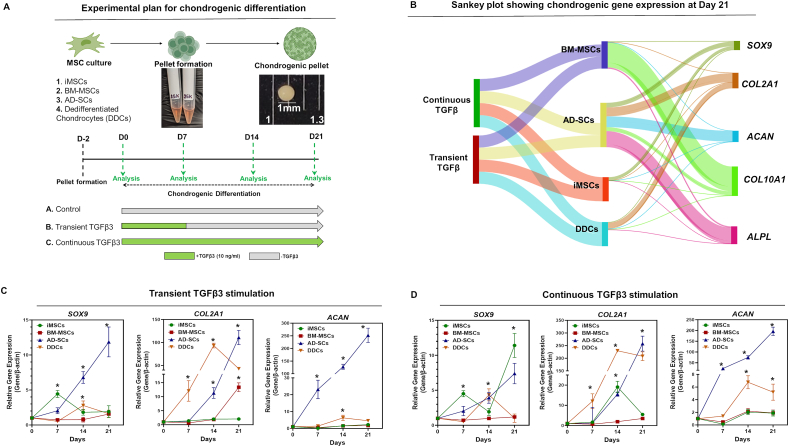
Chondrogenic gene expression during chondrogenic differentiation of iMSCs and adult somatic MSCs. **(A)** Schematic representation of chondrogenic differentiation protocol for iMSCs, BM-MSCs, ADSCs, and DDCs using the pellet culture method. **(B)** The Shankey plot shows the average expression of chondrogenic and hypertrophic genes at day 21 of chondrogenic differentiation during transient and continuous TGFβ3 stimulation across four cell types (*n* = 3 biological replicates). **(C, D)** Quantitative PCR analyses of the relative expression levels of chondrogenic genes (SOX9, COL2A1, ACAN) in pellet cultures at days 7, 14, and 21 under (C) transient and (D) continuous TGFβ3 stimulation across all four cell types. β-actin was used as the housekeeping gene and internal control. Each data point represents the average of three biological replicates (independent donors; *n* = 3) with three technical replicates per time point for each cell type. Data were presented as mean ± standard deviation. Statistical analysis was performed using one-way ANOVA for each cell type, with ∗*p* ≤ 0.05 indicating statistically significant differences at the indicated time points. MSC, mesenchymal stem cell; iMSC, induced pluripotent stem cell-derived MSC; BM-MSC, bone marrow-derived MSC; ADSC, adipose tissue-derived stem cell; DDC, dedifferentiated chondrocyte; TGFβ3, transforming growth factor-beta 3; SOX9, SRY-box transcription factor 9; COL2A1, collagen type II alpha 1; ACAN, aggrecan.

BM-MSCs, ADSCs, DDCs, and iMSCs showed distinct expression patterns of key chondrogenic genes, such as SRY-box transcription factor 9 (*SOX9*), collagen type II alpha 1 (*COL2A1*), and aggrecan (*ACAN*), under these conditions (Fig. 2B–D). All MSC types exhibited a time-dependent increase in the expression of these genes, with levels rising significantly from day 7 to day 21 (Fig. 2C, D). Among adult somatic MSCs, ADSCs demonstrated the highest expression of *SOX9*, *COL2A1*, and *ACAN*, significantly surpassing BM-MSCs (*p* < 0.05). Although donor-to-donor variability in absolute gene expression levels was observed, particularly within the BM-MSC and ADSC groups, such variation is anticipated given the inherent biological heterogeneity of primary human MSCs. Nevertheless, the overall expression trends, particularly the time-dependent increase in key chondrogenic markers, such as SOX9, COL2A1, and ACAN, were consistently observed across donors (Fig. S1, S2; *p* < 0.05, one-way ANOVA). These patterns suggest a degree of consistency in chondrogenic response despite inter-individual differences, supporting the generalizability of our findings. Interestingly, the expression of chondrogenic genes in somatic MSCs under transient TGFβ3 treatment was comparable to continuous stimulation at day 14 and day 21, suggesting that short-term TGFβ3 exposure is sufficient to induce chondrogenesis, consistent with previous findings^47^^,^^49^ (Fig. 2C, D). Notably, ADSCs displayed superior chondrogenic potential compared with BM-MSCs and DDCs, with the overall differentiation hierarchy at day 21 being ADSCs > DDCs > BM-MSCs > iMSCs.

iMSCs, however, responded differently to transient and continuous TGFβ3 stimulation. While continuous TGFβ3 treatment significantly increased SOX9 expression only at day 21, ACAN expression remained low at all time points in both treatment conditions (Fig. 2C, D). This indicates that prolonged TGFβ3 exposure is necessary for effective chondrogenesis in iMSCs. We further assessed chondrocyte hypertrophy by examining the expression of hypertrophic markers alkaline phosphatase (*ALP*) and collagen type X alpha 1 (*COL10A1*). BM-MSCs and ADSCs exhibited higher hypertrophic gene expression than DDCs under both TGFβ3 treatment regimens (Fig. S3). Remarkably, iMSCs showed no detectable expression of *COL10A1* or *ALP* under any condition (Fig. S3; Fig. 2B), suggesting limited hypertrophic differentiation potential. Together, these findings highlight distinct responses of iMSCs and somatic MSCs to TGFβ3 stimulation, with iMSCs requiring prolonged stimulation for effective chondrogenesis.

### Molecular characterization of chondrogenesis reveals distinct signaling mechanisms in iMSCs versus adult somatic MSCs

We identified distinct molecular pathways involved in chondrogenic differentiation for iMSCs compared with BM-MSCs and ADSCs. Signaling pathways and key molecules governing chondrogenic differentiation in iMSCs were akin to those in DDCs (Fig. 3A). While chondrogenic markers like *SOX9* and *COL2A1* exhibited up-regulation across all MSC lines during temporal expression, hypertrophic chondrocyte markers, such as *COL10A1*, *ALP*, and matrix metallopeptidase 13 (*MMP13*), showed increased expression exclusively in BM-MSCs and ADSCs (Fig. 3A). Notably, iMSCs did not express any markers of chondrocyte hypertrophy throughout the chondrogenic differentiation process (Fig. 3A). A focused analysis of signaling pathways involved in MSC chondrogenic differentiation revealed that TGFβ3 stimulation induced the expression of both SMAD2/3 and SMAD1/5 in BM-MSCs and ADSCs. In contrast, TGFβ3 induced the expression of SMAD2/3, but not SMAD1/5, in iMSCs, which may lead to suppressed hypertrophy during chondrogenic differentiation. Our qPCR validation at day 21 chondrogenic culture further showed reduced expression of SMAD1 and SMAD5 in iMSC clones, whereas average expression of these SMADs was significantly higher in multiple donors of BM-MSCs and ADSCs (Fig. S4).

**Figure 3:**
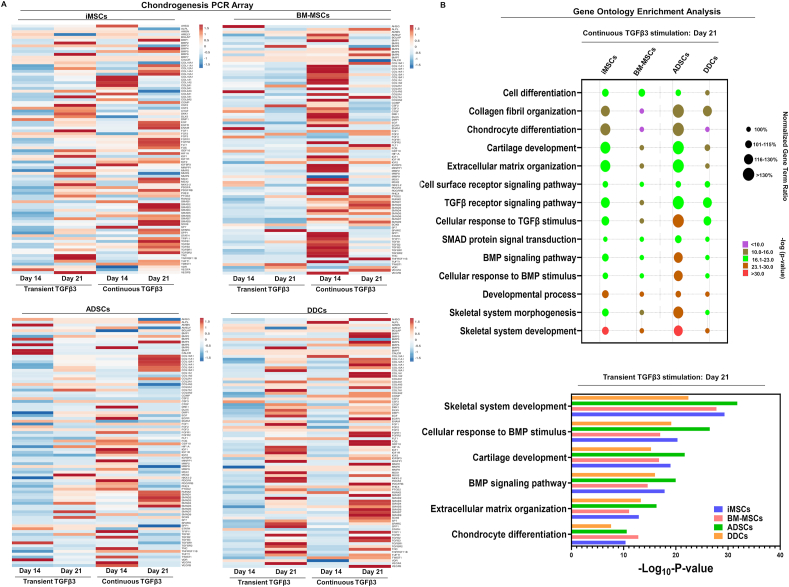
Molecular characterization of MSC chondrogenesis across all four cell types: PCR-based chondrogenic array was performed during chondrogenic differentiation of iMSCs, BM-MSCs, ADSCs, and DDCs. Differential gene expression analyses reveal distinct chondrogenic gene signatures. **(A)** Differentially expressed genes were visualized as a heatmap in all four cell types at day 14 and day 21 of chondrogenic differentiation upon transient and continuous TGFβ3 stimulation. The color key indicates the intensity associated with normalized expression values. Red shades indicate higher expression, and blue shades indicate lower expression. **(B)** Functional annotation clustering using gene ontology analysis for biological processes using differentially expressed genes at day 21 chondrogenic differentiation upon continuous (upper panel) versus transient TGFβ3 stimulation (lower panel). In the upper panel, the Y-axis label represents enriched pathway, the size of the bubble represents the normalized gene term ratio, and the color shows the FDR *p*-value of gene ontology terms. Gene term ratio was calculated by dividing the gene numbers annotated in a pathway by all the gene numbers annotated in this pathway term for each MSC type. Gene term ratio of each cell type was normalized with respect to the MSCs having the lowest strength. MSC, mesenchymal stem cell; iMSC, induced pluripotent stem cell-derived MSC; BM-MSC, bone marrow-derived MSC; ADSC, adipose tissue-derived stem cell; DDC, dedifferentiated chondrocyte; TGFβ3, transforming growth factor-beta 3; FDR, false discovery rate.

Pairwise comparative analysis of DEGs at day 21 identified more than 10 distinct gene clusters in each MSC type under transient and continuous TGFβ3 stimulation. Genes within each cluster were significantly enriched in specific gene ontology terms, such as cartilage development, ECM organization, collagen fibril organization, chondrocyte differentiation, and skeletal system development (Fig. 3B). The gene numbers and significance levels associated with these gene ontology terms varied among iMSCs, BM-MSCs, and DDCs, suggesting differences in cell differentiation and ECM-related genes (Fig. 3B). Subsequent analysis indicated that the differently expressed genes in iMSCs, BM-MSCs, and DDCs function in critical signaling pathways related to TGFβ receptor and BMP signaling, contributing to their distinct chondrogenic responses (Fig. 3B).

### Network analysis identifies distinct GRNs of chondrogenesis in each MSC type

Employing co-expression network analysis, we constructed GRNs to unveil hub genes regulating MSC chondrogenesis. The resulting individual GRNs for each MSC are depicted in Figure 4A**,** where hub genes were identified based on their degree (node connectivity), weight (association between two genes), and betweenness centrality measure within the network. The iMSC chondrogenesis GRN identified *COL2A1*, epidermal growth factor (*EGF*), fibroblast growth factor receptor (*FGFR*), and hypoxia inducible factor-alpha (*HIFA*) as hub genes that regulated chondrocyte differentiation of iMSCs (Fig. 4A). Interestingly, the expression levels of iMSC differentiation hub genes were significantly elevated during TGFβ stimulation in day-21 pellet cultures (Fig. 3A). The iMSCs network module, consisting of 64 genes, was enriched in ECM organization, collagen fibril organization, TGFβ, and insulin-like growth factor complex (Fig. 4B).

**Figure 4:**
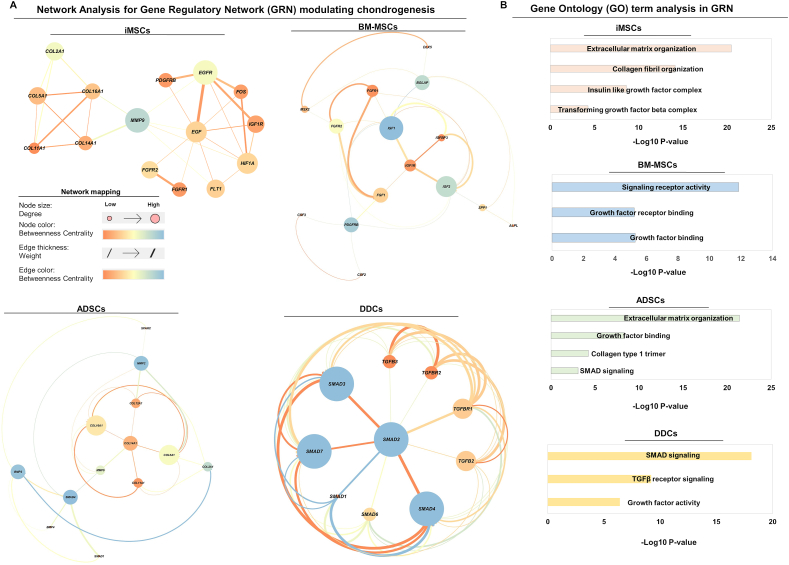
GRNs and associated GO term analysis during chondrogenesis for each MSC type. **(A)** Network analysis for identifying the regulator of chondrogenic differentiation identified a distinct GRN and associated hub gene of chondrogenesis for each MSC type. Gene array data from day 21 pellet cultures under TGFβ3 stimulation were used for modules or clusters for each MSC. **(B)** GO term analysis for enriched biological processes in the GRN of each MSC type. Y-axis label represents enriched GO terms, and *X*-axis label represents negative log of FDR *p*-value. GRN, gene regulatory network; MSC, mesenchymal stem cell; TGFβ3, transforming growth factor-beta 3; GO, gene ontology; FDR, false discovery rate.

Similarly, the BM-MSC network included insulin-like growth factor-1 (*IGF1*), type 1 insulin-like growth factor receptor (*IGF1R*), *FGFR1/2*, and platelet-derived growth factor receptor beta (*PDGFRB*) as hub genes controlling signaling receptor and growth factor binding activity (Fig. 4A, B). The GRN of ADSCs encompassed *SMAD1/4*, *BMP2/4*, collagen type XIV alpha 1 (*COL14A1*), and collagen type XVI alpha 1 (*COL16A1*) as hub genes regulating SMAD signaling, growth factor binding, and ECM organization, contributing to the differentiation of ADSCs into chondrocytes (Fig. 4A, B). Additionally, the GRN of DDCs involved 52 genes related to SMAD signaling, TGFβ receptor signaling, and growth factor activity, with hub genes such as SMAD2/3, SMAD1/4/6/7, and TGFβR1/2 steering chondrogenic differentiation (Fig. 4A, B). Collectively, these findings suggest that GRNs governing chondrogenic differentiation in iMSCs differ distinctly from those in BM-MSCs, ADSCs, and DDCs. Furthermore, the data indicate that these distinct GRNs are fundamental to the varied chondrogenic responses exhibited by each MSC under TGFβ3 stimulation.

### RNA sequencing analysis identifies transcriptomic heterogeneity between iMSCs and adult somatic MSCs

Employing high-depth bulk RNA sequencing on multiple clones of iMSCs and multiple donors of each BM-MSCs, ADSCs, and DDCs, we performed comparative computational analyses, including differential gene expression, gene ontology, hierarchical clustering, principal-component analysis, and network analysis, to unveil the transcriptomic heterogeneity among iMSCs, adult somatic MSCs, and DDCs (Fig. S5). Differential expression analysis indicated a consistent difference in the transcriptome profile among iMSCs, BM-MSC, ADSCs, and DDCs (Fig. 5A). Comparisons of average gene expression between iMSCs and adult MSCs or DDCs unveiled distinct gene expression patterns (Fig. 5B). In total, 4594 genes were differentially expressed between iMSCs and BM-MSCs, 5361 genes between iMSCs and ADSCs, and 7041 genes between iMSCs and DDCs (Fig. 5B). We observed iMSC-specific gene signature featuring Delta and Notch-like epidermal growth factor-related receptor (*DNER*), Toll-like receptor 4 interactor with leucine-rich repeats (*TRIL*), solute carrier family 6 member 15 (*SLC6A15*), SH2 domain containing 3C (*SH2D3C*), *AC117402.1*, Src homology 2 domain containing transforming protein 2 (*SHC2*), actin gamma 2 (*ACTG2*), claudin-1 (*CLDN1*), hematopoietically expressed homeobox (*HHEX*), TGFB2-antisense RNA1 (*TGFB2-AS1*), and myelodysplasia syndrome 1 and ecotropic viral integration site 1 complex locus (*MECOM*) (Fig. 5C). Over 200 distinct genes were specifically expressed in each of the four tested cell types.

**Figure 5:**
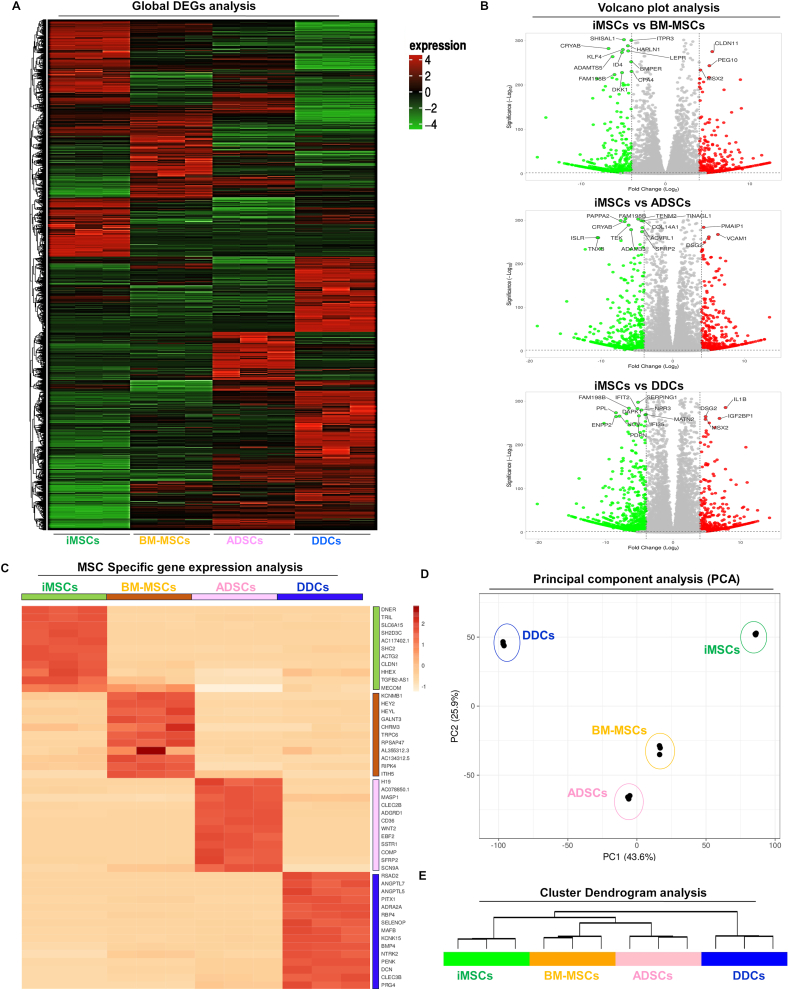
RNA sequencing analysis identifies transcriptomic heterogeneity between iMSCs and adult somatic MSCs: Bulk RNA sequencing was performed for each MSC type at an uncommitted progenitor state without chondrogenic stimulation. Differential gene expression analyses reveal a distinct transcriptomic signature of each MSC population. **(A)** Differentially expressed genes (DEGs) were visualized as a heatmap. Color key indicates the intensity associated with normalized expression values. Red shades indicate higher expression, and green shades indicate lower expression. **(B)** Genes with differential expression levels greater than 4-fold (FDR *p*-value < 0.05) were visualized as a volcano plot showing differential expression profile for each MSC. **(C)** Heatmap of normalized fragments per kilobase of exon per million mapped fragments (FPKM) values showing expression profiles of MSC-specific genes enriched in each cell type. The color key indicates the intensity associated with normalized expression values. Dark color indicates higher expression, and light shades indicate lower expression. **(D)** Principal component analysis using DEGs from transcriptomic datasets for each MSC source (*n* = 3 donors per source), showing segregation of all four cell types. Unit variance scaling was applied to rows; singular value decomposition with imputation was used to calculate principal components. Principal component 1 (*x*-axis) and principal component 2 (*y*-axis) explain 43.6% and 25.9% of the total variance, respectively. **(E)** Cluster dendrogram analysis using significant DEGs (Log fold-change >2; FDR <0.05) between all sources of MSCs. Unit variance scaling was applied to rows. Imputation was used for missing value estimation. Columns were clustered using the correlation distance and average linkage method. MSC, mesenchymal stem cell; iMSC, induced pluripotent stem cell-derived MSC; FDR, false discovery rate.

Unsupervised clustering identified a distinct cluster of DDCs from all MSCs (Fig. 5E). Additionally, clustering distinguished iMSCs from adult somatic MSCs, further dividing the latter into two groups based on tissue of origin, with BM-MSCs in one cluster distinct from ADSCs in another (Fig. 5E). These clustering patterns underscore substantial heterogeneity within MSCs derived from iPSCs, bone marrow, and adipose tissue, corroborating the findings in the global heatmap (Fig. 5B). Principal component analyses of the DEGs resulted in the segregation of iMSCs, BM-MSCs, ADSCs, and DDCs into four distinct clusters (Fig. 5D). This separation suggests unique transcriptome profiles for each of these MSCs.

### Functional heterogeneity identifies divergent cell signaling profiles between iMSCs and adult somatic MSCs

We next assessed the functional consequence of transcriptomic heterogeneity observed between iMSCs and adult somatic MSCs. Our pairwise comparative DEGs between iMSCs *vs*. BM-MSCs, iMSCs *vs*. ADSCs, or iMSCs *vs*. DDCs identified 418 (4.4%) DEGs to be common among iMSCs, BM-MSCs, ADSCs, and DDCs. The distribution of their DEGs is shown in Figure S6. These common MSC DEGs predominantly belonged to gene clusters localized in the plasma membrane (26%), followed by the extracellular region (16%), extracellular exosomes (11%), endoplasmic reticulum (10%), mitochondria (9%), cytoskeleton (7%), synapse (6%), cell surface (5%), cell–cell junction (5%), and ECM (5%) (Fig. S7). The pathways preferentially up-regulated in iMSCs included those exclusively related to ECM remodeling, organization, collagen chain formation, and collagen degradation (Fig. 6A). This enrichment involved numerous ECM proteolytic enzymes, such as matrix metalloproteinases (MMP1, MMP3), a disintegrin metalloproteinase with thrombospondin motif 18 (ADAMTS18), various collagens (COL1A1, COL8A1, COL5A3, COL18A1, COL4A2, COL5A1, COL5A2, COL4A1, COL19A1, COL9A3), and adhesion molecules (PECAM1, CD31), which facilitate cell–cell communication and contribute to the structural framework of the basement membrane. The genes associated with ECM organization further implicated cytoskeletal proteins, including elastin microfibril interfacer 3 (EMILIN3), alpha-actinin (ACTN1), integrin subunit alpha 1 (ITGA1), and intercellular adhesion molecules (ICAM1).

**Figure 6:**
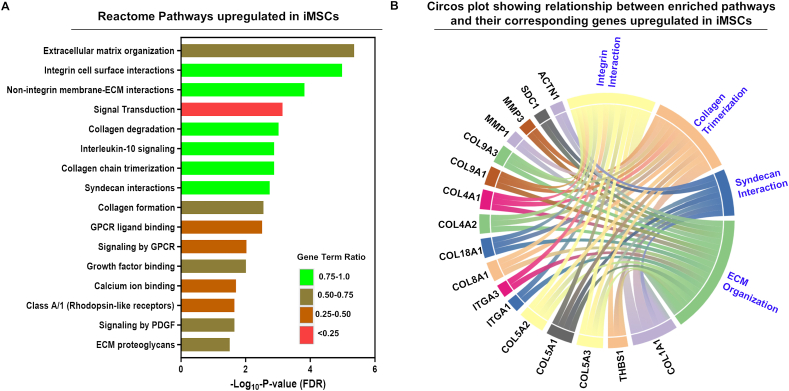
Transcriptomic analysis identifies divergent cell signaling profiles between iMSCs and adult MSCs: Bulk RNA-sequencing data for adult MSCs were analyzed in comparison to iMSCs to identify differentially expressed genes and associated cell signaling pathways. **(A)** All significant (FDR <0.05) Reactome pathways up-regulated in iMSCs compared with BM-MSCs, ADSCs, and DDCs. Y-axis label represents enriched gene ontology terms, and *X*-axis label represents negative log of FDR *p*-value. The color of the bar graph represents the gene term ratio. Gene term ratio was calculated by dividing the gene numbers annotated in a pathway by the gene numbers annotated in this pathway. **(B)** Circos plot of the relationship between enriched cytokine and chemokine pathways and their corresponding genes. Ribbon size encodes the cell value associated with the row/column segment pair. The column segment value and ribbon color are decided by the number of interactions. MSC, mesenchymal stem cell; iMSC, induced pluripotent stem cell-derived MSC; BM-MSC, bone marrow-derived MSC; ADSC, adipose tissue-derived stem cell; DDC, dedifferentiated chondrocyte; FDR, false discovery rate.

Additionally, iMSCs exhibited paracrine signaling involving interleukins and chemotactic cytokines (C-X-C-L) (Fig. 6A). We investigated the relationship between pathways related to enriched ECM organization, collagen fibril, and syndecan interactions (Fig. 6B). The genes implicated in these pathways, such as integrins (*ITGA1*, *ITGA3*), cell adhesion receptors (*SDC1*, *ACTN1*), and collagens (*COL9A1*, *COL9A2*, *COL4A1*, *COL4A2*, *COL5A1*, *COL5A2*, *COL8A1*, *COL1A1*), were identified, highlighting their role in linking the ECM to the cytoskeleton (Fig. 6B). These receptors serve as connectors that integrate external signals, facilitating intercellular communication to uphold the structured collagen fibril matrix and support cytoskeletal organization of iMSCs (Fig. 6B). iMSCs also induced greater expression of several immune mediators, such as CXC motif chemokine ligand 1 (CXCL1), CXCL8, colony-stimulating factor 2 (CSF2), and leukemia inhibitory factor (LIF) that are canonically associated with activating innate immune cells via their cognate C-X-C motif chemokine receptor 2 (CXCR2) receptors (Fig. 6A). Interestingly, iMSCs also promoted signaling through G-protein-coupled receptor (GPCR) and syndecan interaction pathways that were involved in mediating immune functions and cytoskeletal organization (Fig. 6A, B). Our analysis further suggests that iMSCs may respond to growth factor binding, mainly via the activation of platelet-derived growth factor (PDGF) signaling, involving thrombospondin proteins (THBS1, THBS2), and adhesive glycoproteins mediating cell-to-cell and cell-to-matrix interactions (Fig. 6A, B). Altogether, our analyses indicated that iMSCs reveal unique cell signaling patterns potentially involving ECM remodeling, collagen regulation, pro-inflammatory responses, and growth factor signaling, indicative of potential variations in immune modulation and tissue repair capacities.

### RNA-sequencing analysis identifies novel markers enriched in MSCs

Despite clear functional distinctions, the iMSCs and adults somatic MSCs did not differ across classical surface markers, including positive expression of CD29, CD44, CD73, CD90, CD105, and CD166 and no expression of CD11b, CD14, CD31, CD34, CD45, and human leucocyte antigen DR (HLA-DR) (Fig. S8). Consequently, we elected to seek identification of additional markers that not only define iMSCs but also have the potential to capture the functional variability, as well as the biological performance of MSCs. We examined the expression of 393 genes encoding CD markers and related cell surface proteins (including receptors, integrins, and transporters) for iMSCs, BM-MSCs, ADSCs, and DDCs (Fig. 7A). iMSCs expressed 204/393 surface protein-encoding genes, while BM-MSCs, ADSCs, and DDCs expressed 223, 212, and 219 genes, respectively (Fig. 7A). We next filtered the surface protein-encoding genes with expression > 0.1 FPKM and identified 176 core CD genes robustly expressed across four MSCs (Fig. 7A). As expected, commonly known MSC markers (CD29, CD36, CD44, CD73, CD90, CD105, CD106, CD166) were highly expressed across four cell types. The expression profile of these core 176 CD genes indicated distinct expression patterns across iMSCS, BM-MSCs, ADSCs, and DDCs (Fig. 7B). These data also revealed cell-type-specific markers (highlighted by purple squares in the heatmap) (Fig. 7B). Hierarchical clustering of surface markers revealed that iMSCs were more similar to BMSCs than ADSCs (Fig. 7B). Shared genes between iMSCs and BM-MSCs were more abundant than ADSCs and DDCs, and more unique surface genes were detected in ADSCs and BM-MSCs than DDCs (Fig. 7C).

**Figure 7:**
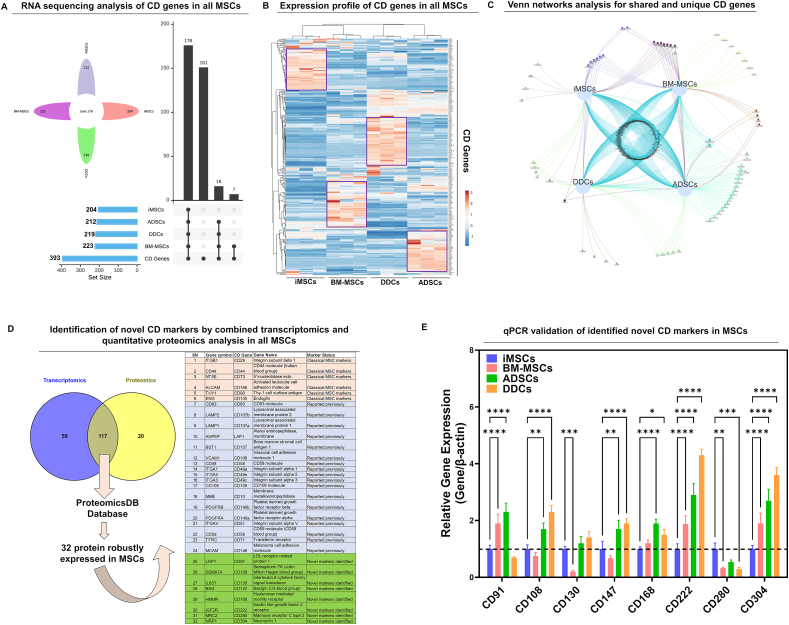
RNA sequencing analysis identifies novel markers enriched in MSCs. **(A)** High-resolution RNA-sequencing analysis for 393 surface marker gene expression in each MSC population. The upset plot shows the number of expressed CD genes in each MSC, and their comparison, identifying 176 core CD genes commonly expressed in all MSCs. **(B)** The expression profile of 176 CD genes was visualized as a heatmap in all four cell types. The genes highlighted in purple indicate specific CD markers enriched in a particular MSC type. The color key indicates the intensity associated with normalized expression values. Red shades indicate higher expression, and blue shades indicate lower expression. **(C)** Venn network analysis showed shared and unique CD genes across all four MSCs. **(D)** Venn diagrams identified MSC surface markers through combined transcriptomics and proteomics analysis for BM-MSCs. The common 117 markers were further compared with CD markers for mesenchymal stem cells available in the ProteomicsDB database, which identified a list of 32 CD markers. The table shows the 32 CD markers, including classical known CD markers (highlighted in orange color), previously reported markers enriched in MSCs (highlighted in blue color), and novel CD markers enriched in MSCs (highlighted in green color). **(E)** Quantitative PCR analysis showed gene expression of CD markers among all four cell types. β-actin served as the housekeeping gene and internal control. Values represent fold induction (mean ± standard deviation) relative to iMSCs. ∗∗∗∗*p* ≤ 0.0001, ∗∗∗*p* ≤ 0.001, and ∗∗*p* ≤ 0.01 indicate that values are statistically significantly different in adult MSCs versus iMSCs; “ns” indicates no statistical significance between comparison groups. MSC, mesenchymal stem cell; iMSC, induced pluripotent stem cell-derived MSC; BM-MSC, bone marrow-derived MSC.

### Integrated proteome-transcriptome analyses further refined novel markers enriched in MSCs

Our integrated analysis revealed that 66% (117/176) of surface marker genes identified through RNA sequencing were validated using publicly available liquid chromatography-mass spectrometry data^45^ (Fig. 7D). To further validate these MSC markers, we cross-referenced the 117 genes with protein expression data from the ProteomicsDB database,^46^ which analyzed BM-MSCs from three male donors (a design differing from our study), which included two male and one female donor. This comparative analysis identified 32 common surface markers designated as “CD markers enriched in MSCs” due to their high abundance in MSCs (Fig. 7D). Despite differences in donor composition, the overlap between the datasets was substantial. It is possible, however, that using gender-matched donor groups might have revealed an even greater number of shared markers. The protein expression profiles of these CD markers were visualized as a heatmap for a broad range of mesenchymal tissues (Fig. S9). High expression levels of thymocyte differentiation antigen 1 (THY-1 or CD90), vascular cell adhesion molecule-1 (VCAM1 or CD106), and low-affinity nerve growth factor receptor (LNGFR or CD271) on MSCs were proposed as a quality control for clinical use of MSCs, as they were associated with self-renewal and robust lineage differentiation. Among these markers enriched in MSCs, 18 CD markers were reported previously to be expressed in BM-MSCs^50^ (Fig. 7D).

Interestingly, our integrated transcriptome-proteome analysis identified eight CD markers that were previously not reported as markers enriched in MSCs. These CD markers included CD91 (low density lipoprotein receptor-related protein 1 or LRP1), CD108 (semaphorin 7A or SEMA7A), CD130 (interleukin 6 cytokine family signal transducer or IL6ST), CD147 (basigin or BSG), CD168 (hyaluronan mediated motility receptor or HMMR), CD222 (insulin-like growth factor 2 receptor or IGF2R), CD280 (mannose receptor C-type 2 or MRC2), and CD304 (neuropilin 1 or NRP1), which we termed as “novel CD markers” enriched in MSCs. We validated the expression of these novel markers at the mRNA level across all four cell types (Fig. 7E). While mRNA levels of classical CD markers do not exhibit significant variation among different MSCs, our novel identified markers exhibited a differential expression pattern in adult MSCs versus iMSCs (Fig. 7E; Fig. S10). Furthermore, there was a lack of consistent pattern in the expression of known markers, and no difference between commonly used markers was observed between all four cell types (Fig. S10). However, a significant difference in expression of the new CD marker was observed, which suggests potential utility of these markers as MSC markers at least in the cell type studied. These identified surface markers were found to be enriched in known functional categories, including cell surface, focal adhesion, receptor complex, cell–cell junction, pigment granule, lysosome, and plasma membrane raft, among highly enriched categories (Fig. S11).

## Discussion

Our study established distinct transcriptional control of chondrogenesis between iMSCs and adult somatic MSCs, revealing a unique mechanism by which iMSCs differentiated towards chondrocytes without undergoing hypertrophy. Using an unbiased genomic and proteomic approach, we proposed a novel and comprehensive surface marker set for MSC characterization, capturing functional and transcriptomic heterogeneity across cell sources, and offering a robust tool to better harness MSC potential. We utilized iMSCs derived from iPSCs, which we previously generated from normal human articular chondrocytes (AC-iPSCs).^14^^,^^28^ Our prior comparative studies demonstrated that AC-iPSCs exhibited significantly higher chondrogenic potential compared with iPSCs derived from skin fibroblasts (SF-iPSCs) or cord blood cells (CB-iPSCs), underscoring the importance of tissue of origin in determining the chondrogenic potential of iPSCs.^28^ The enhanced chondrogenic performance of AC-iMSCs likely stems from their tissue-specific epigenetic landscape, which retains a memory of their original cell type.^14^ However, further studies are needed to fully understand the influence of tissue origin on iPSC behavior and to enhance the generalizability of iMSC-based approaches.

Several studies, focusing on increased mRNA expression of chondrogenic markers^21^ or proteoglycan staining,^21^^,^^51^^,^^52^ highlighted the ability of iMSCs to form cartilage. Kang et al discovered that iMSCs could generate chondrocytes in a similar manner to BM-MSCs.^53^ Additional studies emphasized the robust chondrogenic differentiation of iMSCs.^21^^,^^22^^,^^54^ To directly benchmark iMSCs against currently used MSC populations, we compared the chondrogenic potential of iMSCs with that of adult somatic MSCs, including BM-MSC and ADSCs. Our findings align with these reports, demonstrating the potential of iMSCs to generate hyaline-like cartilage. Yet, we revealed a distinct chondrogenic potential for iMSCs compared with other adult MSCs. Despite requiring a longer duration of TGFβ3 stimulation and expressing a lower level of *COL2A1* and *ACAN*, cartilage derived from iMSCs lacked expression of hypertrophic markers such as *COL10A1* and *ALPL*. Hypertrophy is commonly associated with the chondrogenesis of adult MSCs and has plagued clinical translation of this regenerative approach due to somatic MSCs developing an adverse fibrous phenotype with limited host integration. Our study thus established that iMSC-derived cartilage exhibits phenotypic characteristics akin to hyaline cartilage, holding promise for regenerating *de novo* hyaline cartilage. While our data demonstrated that iMSCs did not exhibit any signs of hypertrophy during the 21-day culture period, we do not exclude the possibility of a delayed hypertrophy that may appear at much later time points. We therefore acknowledge that the restricted culture time to 3 weeks is a potential limitation of this study. Future investigations using extended *in vitro* cultures, *in vivo* models, and functional assays may be required to reveal long-term matrix integrity, functional performance, and potential of iMSC-derived pellets in resurfacing focal chondral defects in rats and large animal models.

RUNX2 is a crucial regulator of hypertrophy,^55^^,^^56^ as suppressing RUNX2 in MSCs significantly reduces COL10 expression after chondrogenesis.^57^ In our study, we observed significantly reduced *RUNX2* expression during iMSC chondrogenesis at days 14 and 21 compared with BM-MSCs or ADSCs. Following TGFβ3 stimulation, iMSCs did not express RUNX2, suggesting that TGFβ3 may not activate the hypertrophic phenotype in iMSCs. Our results were in line with previous reports demonstrating lower RUNX2 expression in naïve iPSCs compared with undifferentiated MSCs.^58^ These results collectively indicate that iMSCs inherently express lower levels of RUNX2, thereby limiting hypertrophic and osteogenic transition during chondrogenesis.

We then investigated the activation of SMAD2/3 and SMAD1/5. SMAD2/3 activation is necessary to induce chondrogenesis in MSCs by activating SOX9, a key factor in early chondrogenesis.^59^ Conversely, Smad3 knockdown significantly inhibits TGFβ-induced chondrogenic differentiation.^60^ Interestingly, our results indicated that TGFβ3 stimulation of iMSCs led to higher SMAD2/3 expression, potentially increasing SOX9 expression and inducing chondrogenesis. Notably, P-Smad1/5 is considered a key signaling pathway leading to chondrocyte hypertrophy.^56^^,^^61^ In our study, TGFβ3 addition did not induce SMAD1/5 expression in iMSCs. However, robust activation of SMAD1/5 was observed in adult somatic MSCs. Therefore, reduced SMAD1/5 expression may be associated with the lack of hypertrophic induction in iMSCs compared with BM-MSCs and ADSCs. To the best of our knowledge, no studies have reported how TGFβ-Smad and BMP-Smad signaling pathways interact during iMSC chondrogenesis and hypertrophy. Our current findings suggest that the temporal activation of Smad1/5 signaling during chondrogenic differentiation may contribute to the generation of hyaline cartilage in iMSCs.

Our study makes a significant contribution by constructing the gene regulatory network for iMSC chondrogenesis, ensuring that the identified hub genes truly govern chondrogenic differentiation. In addition to conventional master transcription factors like SOX9, we identified several additional hub genes associated with iMSC chondrogenesis. Notably, the expression levels of *EGF*, *FGFR1*, *FGFR2*, *FLT1*, *IGF1R*, and *HIF1A* were strongly correlated with chondrogenic genes. HIF-1α and HIF-2α are primary HIF-familial factors known to promote chondrogenesis, with recent studies demonstrating their role in promoting the expression of COL2A1, SOX9, and ACAN, while inhibiting fibroblastic markers.^62^ Emerging evidence suggests the involvement of FGFs in chondrocyte differentiation and maturation by boosting ECM production. Our study identified IGF1, IGF2, and IGF1R as hub genes in the gene regulatory network of BM-MSC chondrogenesis, noting that IGFs were enriched in the chondrogenic pellets at day 21 of chondrogenic differentiation. These findings align with previously established roles for IGFs in promoting chondrocyte development.^63^^,^^64^

The plasticity of mesenchymal progenitors is crucial for applications in cellular therapy and tissue engineering. Presently, there is no standardized guideline for characterizing MSCs; consequently, the minimal criteria, including surface markers as described by the ISCT, are utilized to define MSCs.^30^ In this investigation, we conducted an integrated analysis of deep sequencing data at the genomic and proteomic levels to delineate the surfaceome profile of MSCs from our four different sources. We identified and validated several non-classical MSC markers, potentially aiding in the further characterization of somatic MSCs (BMSC and ADSCs) and iMSCs. Our refinement method using a combination of transcriptomic and proteomic analyses revealed eight CD markers across all four MSC types, the regulation of which may predict the chondrogenic potential of each MSC source. Although our study included BM-MSC and ADSC donors of mixed sexes, and iMSC clones were derived from a male donor cell line, the ProteomicsDB dataset used for validating protein-level expression of surface markers was derived from male BM-MSC donors. This sex mismatch may introduce bias in comparative proteomic analyses. While the primary goal of our marker discovery approach was to identify broadly enriched CD markers across cell types, future studies are warranted to explore sex-specific differences in MSC biology, including chondrogenic capacity and immunomodulatory function. Our study further provides a comprehensive understanding of MSC behavior, emphasizing the importance of origin-specific considerations and the potential of iMSCs for regenerative therapies. The exploration of surface markers, gene regulatory networks, and functional assessments contributes valuable insights into refining molecular descriptions and standardizing MSCs for therapeutic applications.

## CRediT authorship contribution statement

**Nazir M. Khan:** Writing – original draft, Visualization, Validation, Methodology, Investigation, Formal analysis, Data curation, Conceptualization. **Thanh N. Doan:** Writing – review & editing, Software, Methodology, Investigation, Formal analysis, Data curation. **Jarred M. Kaiser:** Writing – review & editing, Validation, Methodology, Investigation. **Hicham Drissi:** Writing – review & editing, Supervision, Resources, Project administration, Funding acquisition, Conceptualization.

## Data availability

The RNA-sequencing data generated and analyzed in the current study will be made available upon acceptance in the Gene Expression Omnibus (GEO) database with specific accession number.

## Funding

This work was supported by Veteran Affairs CaReAP Award (USA) (No. I01-BX004878), Veteran Affairs Merit Award (USA) (No. I01-BX004708), and Veteran Affairs CReATE Motion Award (USA) (No. I50-RX004845) to HD.

## Conflict of interests

Hicham Drissi is the Associate Editor of *Genes & Diseases*, but he/she has no involvement in the peer-review of this article and has no access to information regarding its peer-review. The rest authors declared no conflict of interests.

## Figures and Tables

**figs1:**
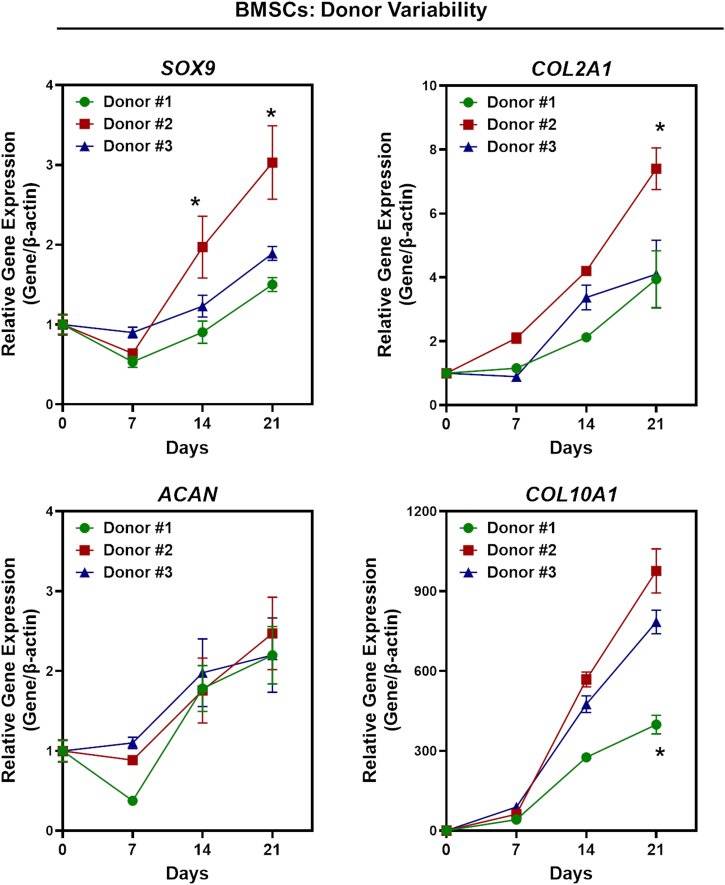


**figs2:**
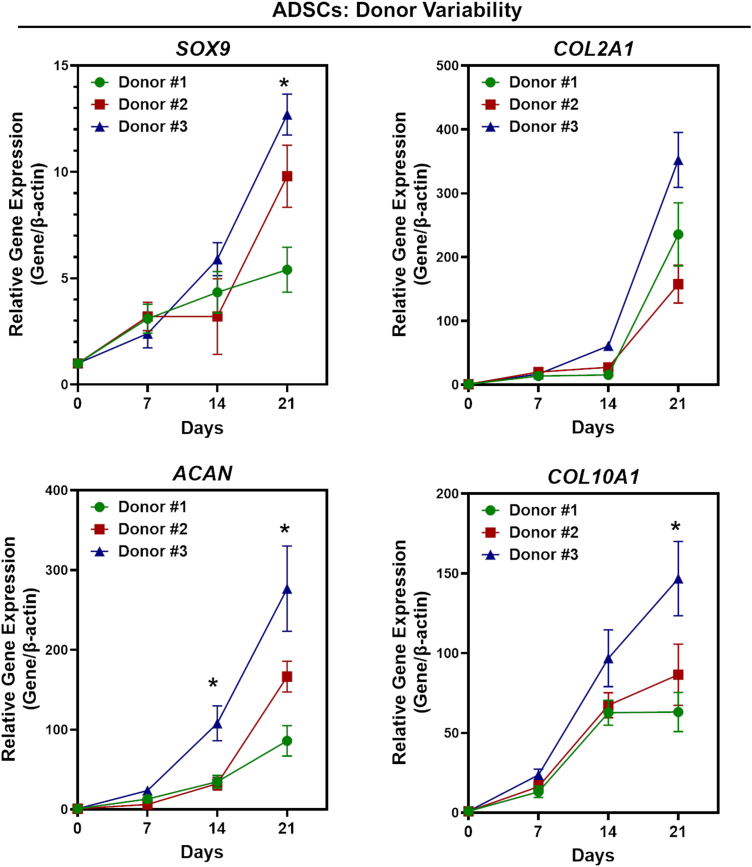


**figs3:**
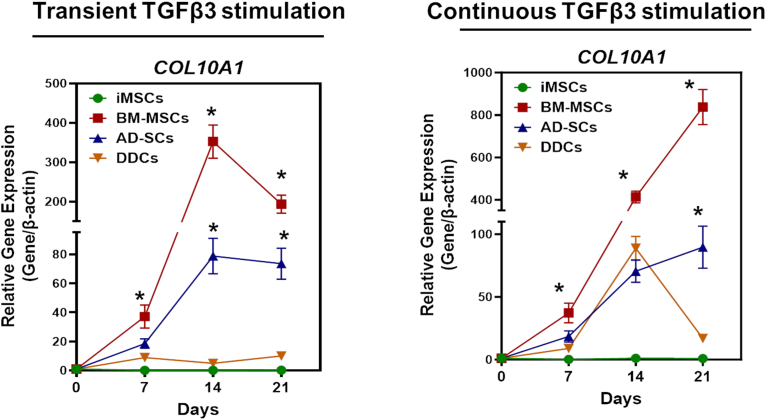


**figs4:**
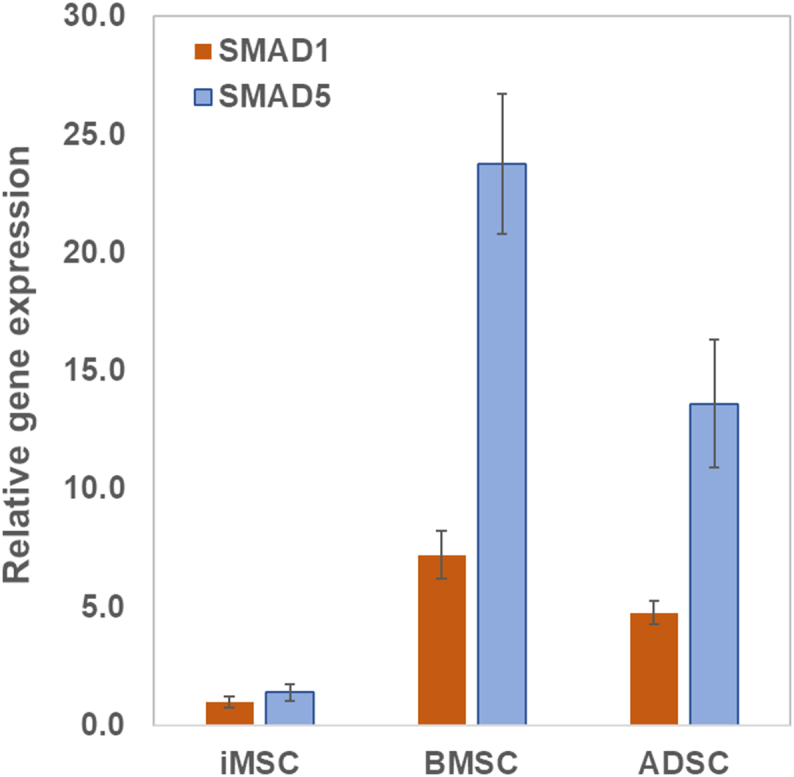


**figs5:**
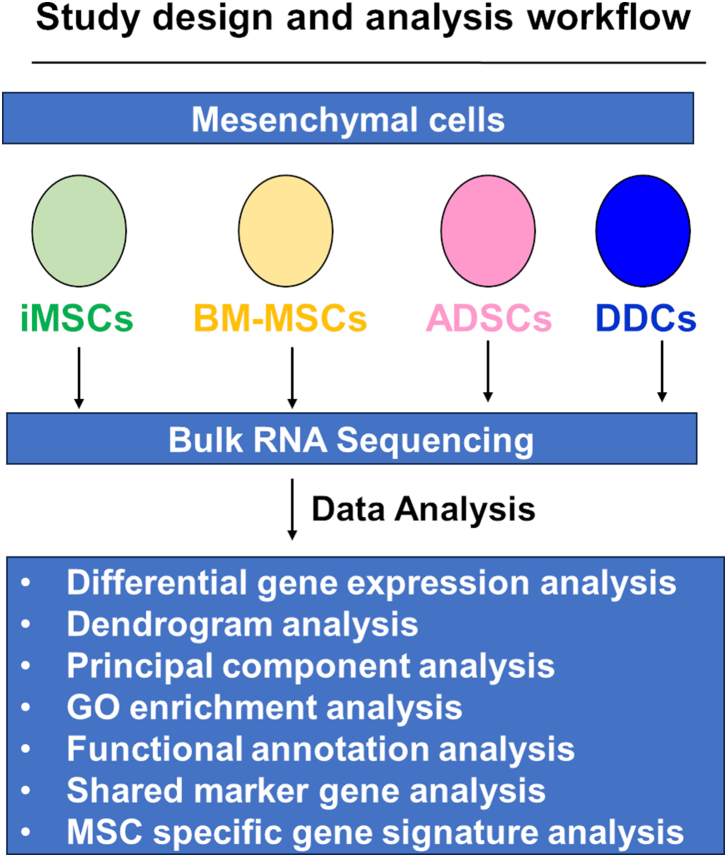


**figs6:**
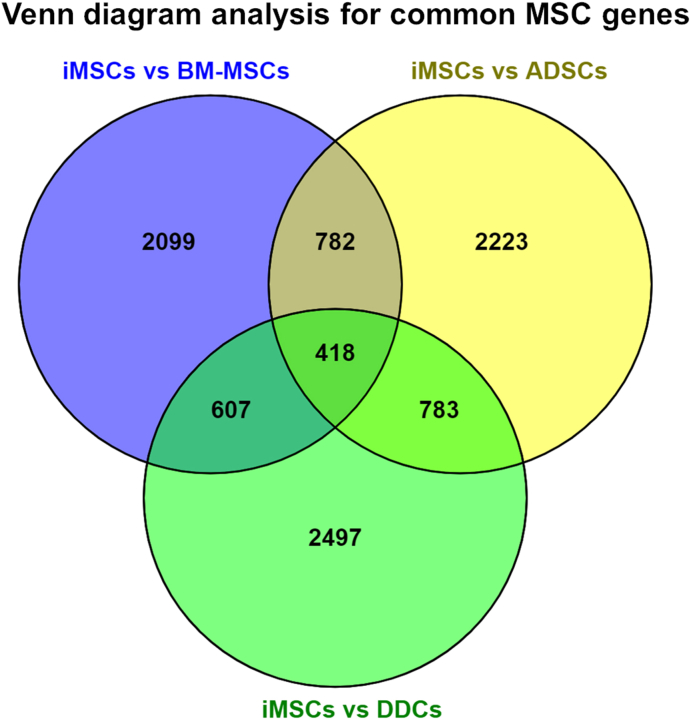


**figs7:**
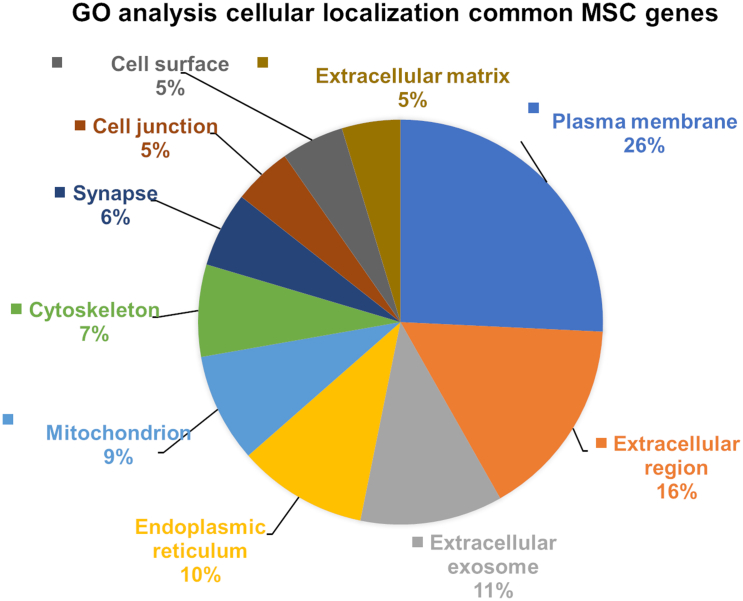


**figs8:**
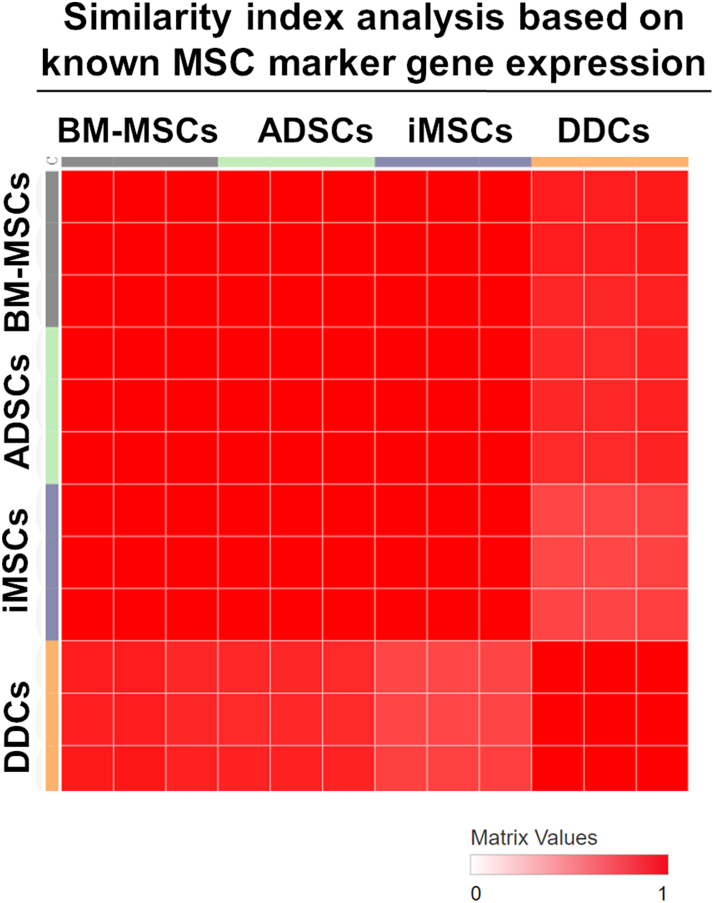


**figs9:**
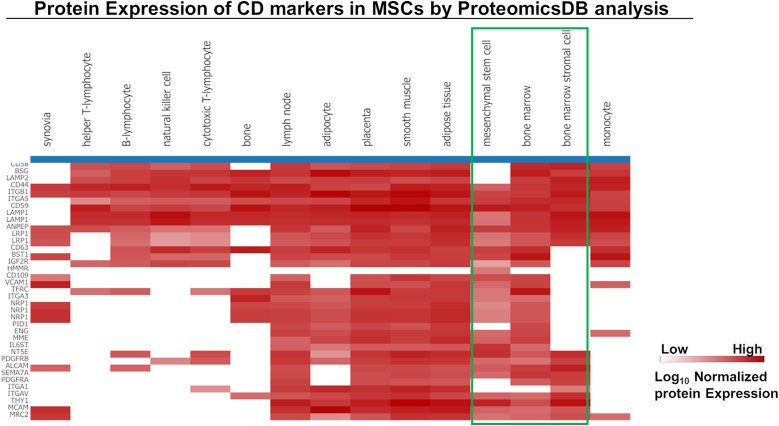


**figs10:**
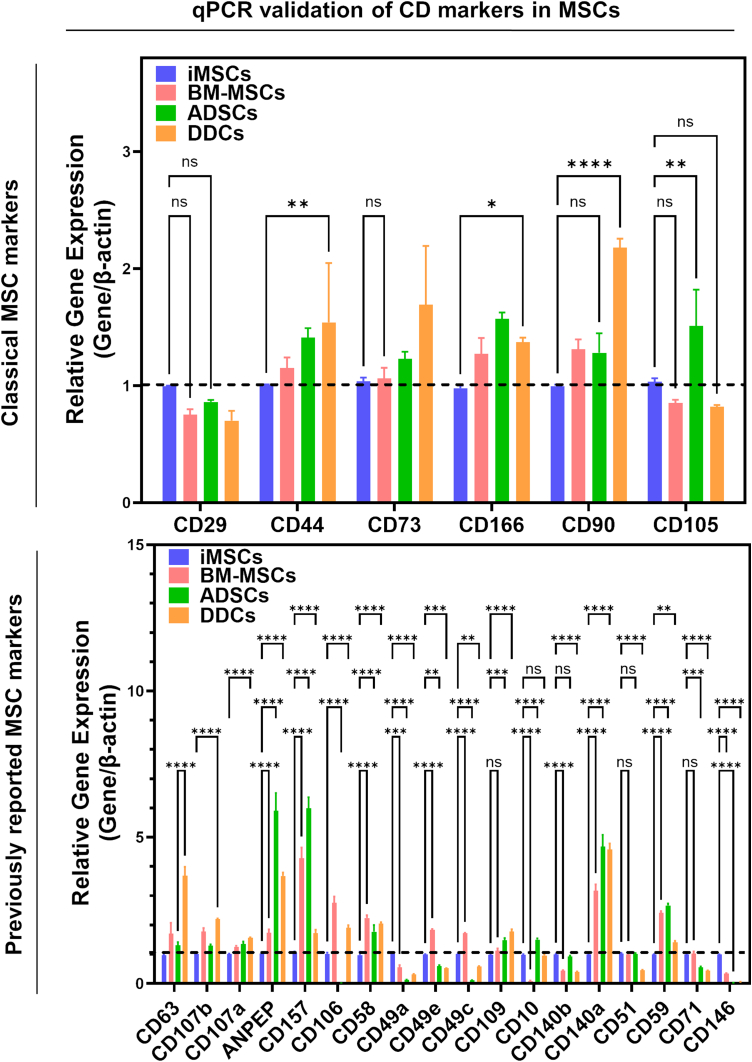


**figs11:**