# Supplementary material for: Chondrogenic potential of mesenchymal progenitors from somatic and cartilage-derived iPSCs is predicted by their transcriptomic signatures
Source: Genes Dis. 2025 Jun 22;13(2):101730. doi: 10.1016/j.gendis.2025.101730 (PMC12765249; doi:10.1016/j.gendis.2025.101730)
Supplement: Multimedia component 1 [file mmc1.docx]

**Figure S1** Quantitative PCR analyses of chondrogenic genes (*SOX9*, *COL2A1*, and *ACAN*) and hypertrophy gene (*COL10A1*) in day 7, 14, and 21 pellet culture upon continuous TGFβ3 stimulation in individual donors (#1, #2, #3) of BM-MSCs. Values represent fold induction (mean ± standard deviation) relative to the levels at day 0 for each MSC donor. The data point for each biological donor represents the average of three technical replicates per time point. Statistical analysis was performed using one-way ANOVA for each cell type, with ^*^*P* ≤ 0.05 indicating statistically significant differences in each donor at the indicated time point. BM-MSC, bone marrow-derived MSC; TGFβ3, transforming growth factor-beta 3; SOX9, SRY-box transcription factor 9; COL2A1, collagen type II alpha 1; ACAN, aggrecan; COL10A1, collagen type X alpha 1.

**Figure S2** Quantitative PCR analyses of chondrogenic genes (*SOX9*, *COL2A1*, and *ACAN*) and hypertrophy gene (*COL10A1*) in day 7, 14, and 21 pellet culture upon continuous TGFβ3 stimulation in individual donors (#1, #2, #3) of ADSCs. Values represent fold induction (mean ± standard deviation) relative to the levels at day 0 for each MSC donor. The data point for each biological donor represents the average of three technical replicates per time point. Statistical analysis was performed using one-way ANOVA for each cell type, with ^*^*P* ≤ 0.05 indicating statistically significant differences in each donor at the indicated time point. ADSC, adipose tissue-derived stem cell; TGFβ3, transforming growth factor-beta 3; SOX9, SRY-box transcription factor 9; COL2A1, collagen type II alpha 1; ACAN, aggrecan; COL10A1, collagen type X alpha 1.

**Figure S3** Quantitative PCR analyses of the relative expression levels of COL10A1 in pellet cultures at days 7, 14, and 21 under transient and continuous TGFβ3 stimulation across all four cell types. β-actin was used as the housekeeping gene and internal control. Each data point represents the average of three biological replicates (independent donors; *n* = 3) with three technical replicates per time point for each cell type. Data were presented as mean ± standard deviation. Statistical analysis was performed using one-way ANOVA for each cell type, with ^*^*P* ≤ 0.05 indicating statistically significant differences at the indicated time points. TGFβ3, transforming growth factor-beta 3; COL10A1, collagen type X alpha 1.

**Figure S4** Quantitative PCR analyses *SMAD1* and *SMAD5* in day 21 pellet culture upon continuous TGFβ3 stimulation in ADSCs and BM-MSCs, upon comparison to iMSCs. MSC, mesenchymal stem cell; iMSC, induced pluripotent stem cell-derived MSC; BM-MSC, bone marrow-derived MSC; ADSC, adipose tissue-derived stem cell; TGFβ3, transforming growth factor-beta 3.

**Figure S5** Study design and analysis workflow for transcriptomic analysis in each cell type showing transcriptomic heterogeneity of mesenchymal stem cells (MSCs).

**Figure S6** The Venn diagram showing the distribution of 418 differentially expressed genes (DEGs) overlapped between adult MSCs and DDCs in comparison to iMSCs, as analyzed by Bulk RNA-sequencing analysis for adult MSCs in comparison to iMSCs to identify DEGs and associated cell signaling pathways. MSC, mesenchymal stem cell; iMSC, induced pluripotent stem cell-derived MSC; DDC, dedifferentiated chondrocyte.

**Figure S7** Gene ontology enrichment analysis of common MSC genes indicates the cellular localization of the 418 common differentially expressed genes (DEGs).

**Figure S8** Correlation heatmap analysis between all four cell types based on gene expression profile of known MSC markers, including CD29, CD44, CD73, CD90, CD105, CD166, CD11b, CD14, CD31, CD34, CD45, and HLA-DR. MSC, mesenchymal stem cell; HLA-DR, human leucocyte antigen DR; CD, cluster of differentiation.

**Figure S9** CD markers found to be expressed in MSC by combined RNA-sequencing and proteomic analysis. Their normalized log10 protein expression levels are shown across a wide range of mesenchymal tissues according to ProteomicsDB. MSC, mesenchymal stem cell.

**Figure S10** Quantitative PCR analysis showed the gene expression of classical and previously reported CD markers among all four cell types. β-actin served as the housekeeping gene and internal control. Values represent fold induction (mean ± standard deviation) relative to iMSCs. ^****^*P* ≤ 0.0001, ^***^*P* ≤ 0.001, and ^**^*P* ≤ 0.01 indicate that values are statistically significantly different in adult MSCs as compared with iMSCs; "ns" indicates no statistical significance between comparison groups. MSC, mesenchymal stem cell; iMSC, induced pluripotent stem cell-derived MSC; CD, cluster of differentiation.

**Figure S11** Functional annotation analysis of identified CD markers for enriched gene ontology terms for cellular components using g:Profiler analysis. CD, cluster of differentiation.
